# Risk Stratification of Thyroid Nodules with Bethesda III Category: The Experience of a Territorial Healthcare Hospital

**DOI:** 10.7759/cureus.8202

**Published:** 2020-05-19

**Authors:** Mohamed Al Dawish, Asirvatham Alwin Robert, Khalid Al Shehri, Salwa Hawsawi, Muhammad Mujammami, Ibrahim Ali Al Basha, Mohannad Alrasheed, Shuaa Asiri, Muneerah Alzouman, Eyad Alkharashi

**Affiliations:** 1 Department of Endocrinology, Prince Sultan Military Medical City, Riyadh, SAU; 2 Division of Endocrinology and Metabolism, Department of Medicine, King Saud University, Riyadh, SAU; 3 Department of Radiology and Medical Imaging, Prince Sultan Miltary Medical City, Riyadh, SAU; 4 Department of Radiology and Medical Imaging, Prince Sultan Military Medical City, Riyadh, SAU; 5 Department of Pathology, Prince Sultan Military Medical City, Riyadh, SAU; 6 Department of Endocrine Surgery, Prince Sultan Military Medical City, Riyadh, SAU

**Keywords:** aus/flus, bethesda, thyroid nodules, risk of malignancy, ti-rads, tsh

## Abstract

Background

The Bethesda System for Reporting Thyroid Cytolopathology (TBSRTC) is the standardized category-based reporting system for thyroid nodule (TN) aspirations; however, atypia of undetermined significance/follicular lesion of undetermined significance (Bethesda category III, AUS/FLUS) is the most controversial category. The aim of this study was to identify the degree of malignancy risk and the related risk factors in the surgical pathology of the Bethesda Category III thyroid nodules.

Methods

A total of 4074 patients (15-90 years, 81.5% of females) were subjected to retrospective analysis, and a total of 463 nodules were classified as Bethesda Class III and included in the analysis. Once all the thyroid cytopathological slides and ultrasound (US) reports were reviewed, they were classified according to the Bethesda System for Reporting Thyroid Cytology, the American College of Radiology (ACR) and the Thyroid Imaging Reporting and Data System (TI-RADS).

Results

Among the 463 Bethesda class III nodules, 167 nodules were surgically excised, showing an overall malignancy of 27.6% (n = 46/167). Patients having thyroid-stimulating hormone (TSH) levels of >4.5 mIU/L (35%), TN <2 cm (34.6%), solid or nearly solid (28.7%), highly hypoechoic (58.3%), longer than wide (50%), lobulated (45.5%), punctate echogenic (48.6%), ACR TI-RAD 5 (55.2%) and falling under the ATA category of high suspicion (50%), displayed a higher risk of malignancy (ROM). The chi-square test revealed a strong association between the echogenicity, echogenic foci, ACR TI-RAD and American Thyroid Association (ATA) category between the malignant and benign nodules. The papillary thyroid carcinoma (PTC) follicular variant (39%) and PTC classical (27%) were identified, in this study population, as the commonest forms of thyroid cancer.

Conclusion

The nodules with AUS/FLUS cytology malignancy rate are comparable with the earlier estimations of other countries. The ACR TI-RAD displayed more accurate diagnostic performances in predicting malignancy in the Bethesda III nodules. However, to confirm the accuracy of the molecular marker tests in specific cytological scenarios, more extensive studies are required in the future.

## Introduction

Thyroid nodules (TNs) are common entities, often identified in clinical practice, either during physical examination or incidentally, during different imaging procedures. The TNs have clinical significance, particularly because of their potential for malignancy. In fact, TNs constitute the most common endocrine malignancy. The incidence of thyroid cancer has been globally estimated at around 298,102 new cases, which is 2.1% of all cancers. Females have three times higher incidence rate of thyroid cancer than do males [1].

In recent times, it has become generally accepted that to manage thyroid cancer, multidisciplinary teams in specialized units, adopting evidence-based guidelines, are required. According to the American Thyroid Association (ATA), the guidelines recommended for managing the treatment of thyroid cancer patients, based on the correlation of clinical and sonographic data, include (a) observation, (b) repeated fine-needle aspiration once in every three months, (c) diagnostic surgery, or (d) genetic testing) [2]. The gold standard for TN investigation is fine-needle aspiration cytology (FNAC), and the literature reports show FNA sensitivity varying from 65 to 99%, while the specificity varies from 72 to 100% [3]. These values are strongly dependent on examiner experience and other technical details [3]. The FNAC has been extensively employed to diagnose thyroid carcinoma and has proven to be reliable in preoperative diagnosis. However, FNAC fails to give definitive diagnosis when the results of the Bethesda System for Reporting Thyroid Cytopathology (BSRTC) are indeterminate for categories, such as in the case of atypia of undetermined significance/follicular lesion of undetermined significance (AUS/FLUS) [4].

The Bethesda system accepted to report thyroid cytopathology standardized the reports of TN aspirations; however, the category that attracted the greatest controversy was AUS/FLUS (Bethesda category III) [3,5]. This occurred because follicular lesions without nuclear atypia (i.e., FLUSs) and lesions with nuclear atypia were both integrated into a follicular or a non-follicular pattern (i.e., AUS) [3,5]. In studies done prior, the AUS/FLUS category in FNAC has been reported to pose the greatest challenge; while the malignancy risk in AUS/FLUS has been estimated in BSRTC to hover between 5% and 15%, institutional data have revealed wide variations [6]. It is noteworthy here, however, that recent studies noted values in the 6% to 48% range [7]. The emergence and the U.S. Food and Drug Administration (FDA) approval of molecular testing also hints that this percentage and its associated recommendation require re-examination.

The swift escalation in the rate of thyroid cancer incidence in Saudi Arabia has been reported over the past many years. The National Cancer Registry revealed data indicative of a notable rise in the occurrence of this disease [8]. Among females, thyroid cancer ranks as the second most frequently occurring cancer and as eighth among males, in Saudi Arabia [8]. However, in comparison with the developed countries, only very limited research has been done on the malignancy risks in TNs in Saudi Arabia, as appropriate studies in these specific areas are limited. To the best of our knowledge, this is the first study conducted over a long duration (eight years) to identify the malignancy risk and related risk factors in the surgical pathology of the nodules classified under Bethesda Category III in Saudi Arabia.

## Materials and methods

In this study, performed from January 2011 to December 2018, we used ultrasound (US)-guided FNA to retrospectively review 4850 thyroid nodules (4074 patients), at the Prince Sultan Military Medical City (PSMMC), Riyadh, Saudi Arabia. Among these, the 465 nodules classified as Bethesda class III were included in our analysis. This study done on all patients coming from different regions of Saudi Arabia who were referred to PSMMC gave a good overall representation. Approval for the study protocol was given by the Research and Ethics Committee of PSMMC (#745), Riyadh, Saudi Arabia.

Data collection

Using the patients’ medical charts and cytopathology reports, data was drawn with respect to age, sex, thyroid-stimulating hormone (TSH) levels, cytological features, US reports of the TN, and histological types of the study population. After reviewing the cytopathological slides of the thyroid, classification was done according to the BSRTC system. The TN features drawn from the US images were reviewed in patients who had been subjected to surgery. The clear US images facilitated the TN classification based on the American College Radiology Thyroid Imaging and Reporting Data System (ACR TI-RADS). The US findings were used to assess the TN dimensions.

Thyroid-stimulating hormone levels

The serum TSH levels (normal range: 0.4-4.5 mIU/L) for all the samples were assessed employing the electrochemiluminescence immunoassay technique (Roche Corporation, Indianapolis, IN, USA) [9].

Bethesda system

Currently, the various US-FNAC thyroid specimens are distinguished using the BSRTC system. According to Cibas. this system was introduced in 2009 and has 6 categories as listed: (I) Unsatisfactory (UNS) or nondiagnostic (ND), (II) Benign and nonneoplastic, (III) AUS/FLUS, (IV) Follicular neoplasm or suspicious for follicular neoplasm (FN/SFN), (V) Suspicious for malignancy (SM) but not diagnostic, and (VI) Malignant [10]. Of the five well-experienced interventional radiologists, only one conducted all the FNAs done under US guidance, using 25-gauge needles, with 3 to 5 passes. On-site staining of the FNA was done applying the Diff-Quik stain, and all the samples were tested by the adequacy assessment. All the slides were interpreted by five accredited cytopathologists. From the FLUS, the characteristic small follicular pattern was evident with weakly attached cells and a little quantity of colloid. The AUS nodules were distinctive due to the presence of cellular and nuclear atypia, irrespective of the cell distribution pattern, which could be either follicular, papillary, or non-characteristic [10].

Thyroid Imaging Reporting and Data System

**Figure 1:**
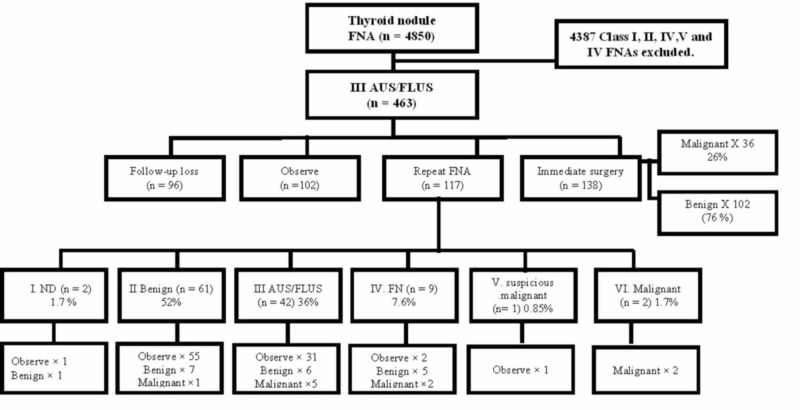
Schematic of the clinical course of AUS/FLUS nodule patients n, the number of thyroid nodules; AUS/FLUS, atypia of undetermined significance/follicular lesion of undetermined significance; FNA, fine-needle aspiration; ND, non-diagnostic; FN, follicular neoplasm

The picture archiving and communication system/radiology information system was used to store the available US images of those patients who had received surgical intervention. The same radiologist with wide experience in thyroid imaging reviewed and described the various TNs, based on the ACR TI-RADS classification. In the ACR TI-RADS, the ultrasound features for malignancy are classified as follows: benign, minimally suspicious, moderately suspicious, or highly suspicious. Points are allotted for all the ultrasound characteristics in a nodule, with additional points being allotted to the more suspicious features. In Figure 1, these characteristics are listed according to the five ultrasound lexicon categories.

 

When a nodule is assessed, the reader chooses one characteristic from each of the first four categories, as well as all the features relevant to the final category and sums up the points. The sum total of the points indicates the ACR TI-RADS level of the nodule, which can range from TR1 (benign) to TR5 (high suspicion of malignancy). It must be remembered that while sometimes a nodule may be found to have zero points (and thus be identified as TR1), all the other nodules will be attributed a minimum of two points because a nodule composed of a mixture of cystic and solid composition (one point) will also merit at least one more point because its solid component will reveal echogenicity.

US and US-guided FNA cytology

The Logiq E9 instrument was employed to perform the US examinations (GE Medical Systems, Milwaukee, WI, USA; 6-15 MHz linear transducer), as well as the Siemens S2000 (Siemens Medical Solutions, Mountain View, CA, USA; 5-14 MHz linear transducer), or IU22 (Philips Medical Systems, Bothell, WA, USA; 5-12 MHz linear transducer) tools. One of the five radiologists having three or more years of experience in thyroid imaging performed the task. 

Image interpretation

Two independent radiologists, different from the ones who did the US imaging retrospectively, interpreted the US characteristics of the TNs. Both radiologists were blinded to the final cytology findings (one radiologist had five years of experience in thyroid US imaging, while the other had three years of experience).

Histological examination

Based on the histological examination, the TNs were classified under benign and nonneoplastic and malignant. In the case of papillary thyroid carcinoma (PTC), the subtype variants were distinguished as follicular, classical, conventional, and tall cell variants. All the patients having the encapsulated follicular variant of papillary thyroid carcinoma (EFVPTC) were once again evaluated and reclassified under a new term “noninvasive follicular thyroid neoplasm with papillary-like nuclear features” (NIFTP). Follicular thyroid carcinoma (FTC) was further distinguished by the terms “widely invasive FTC”, and “minimally invasive FTC”.

In keeping with their histological characteristics, malignancies were subdivided into three groups. The low-risk cancers were labeled completely excised, intrathyroidal, T1-T2 differentiated thyroid carcinomas, lacking vascular invasion (<4 foci were identified for the minimally invasive follicular thyroid carcinomas, FTCs), clinical NO (in patients with ≤ lymph node metastases, all ≤2mm), and in the absence of distant metastasis, all ≤mm), and those lacking distant metastasis. The high-risk cancers, on the other hand, were differentiated thyroid carcinomas exhibiting any one of the following features: gross extrathyroidal extension, lymph node metastases >1 cm or in the lateral compartments, or distant metastases. While high-risk cancers also included medullary thyroid carcinomas, all the other thyroid malignancies were accepted as intermediate [2]. 

Statistical analysis

For all the statistical analyses, the Microsoft Excel 2010 program (Microsoft Corporation, Seattle, WA, USA) and IBM SPSS Statistics (IBM SPSS Statistics for Windows, Version 22, SPSS Inc., an IBM Company) were employed. Frequency and percentages and mean ± SD were used for the descriptive analysis of the epidemiological data. The chi-square test was used to determine the significant difference between the malignant and benign nodules. The risk estimates (odds ratio [OR]) were ascertained and recorded using 95% confidence interval. A P-value of <0.05 was selected to indicate statistical significance.

## Results

The flow chart illustrating the clinical course of the AUS/FLUS nodule in patients is evident in Figure 1.

**Figure 2:**
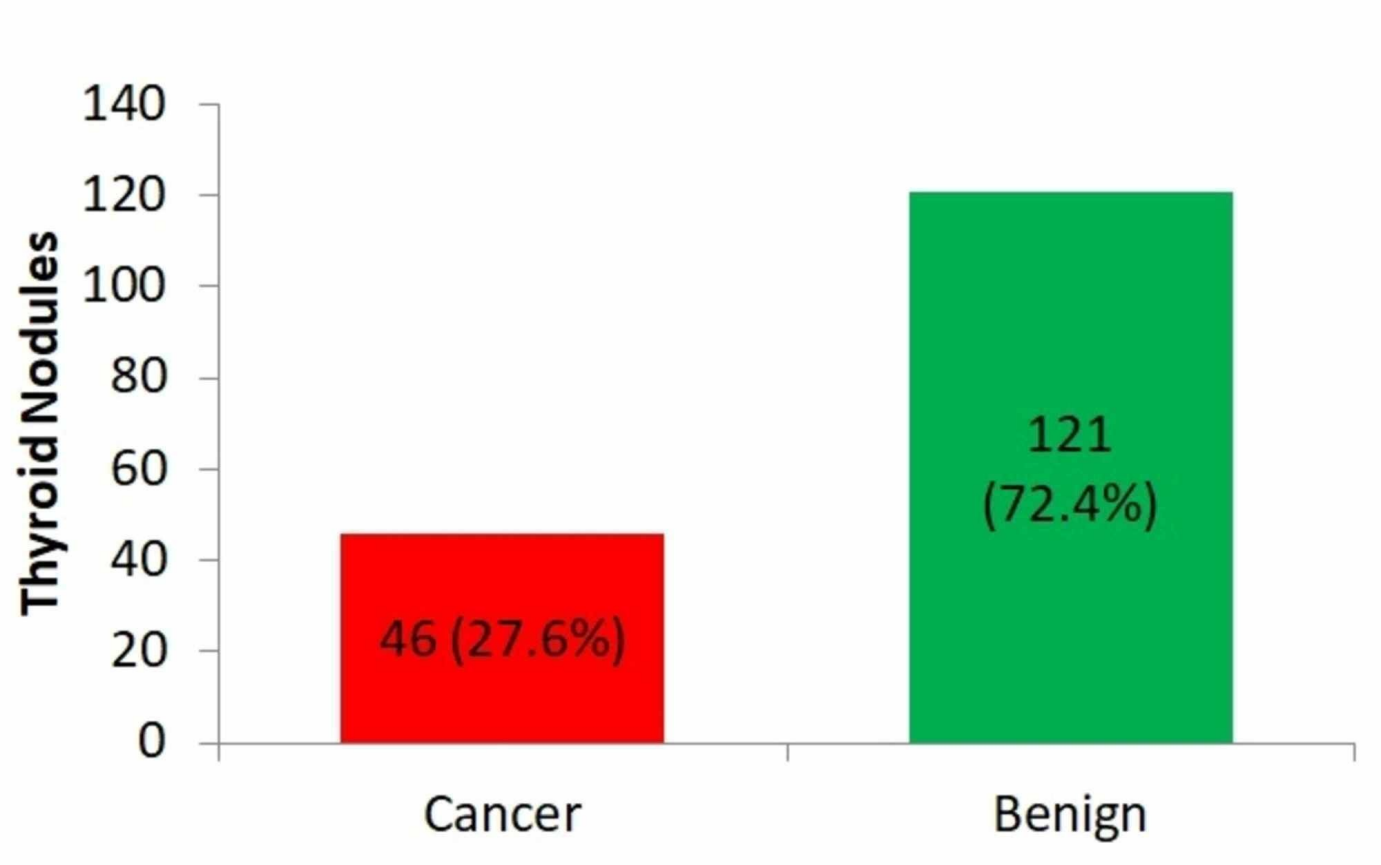
Overall malignancy among the surgical pathology Values are presented as numbers and percentages.

From the 4850 TNs in total, 463 Bethesda class III (81.5% females) nodules were examined in this study; however, the remaining 4387 (Bethesda class I, II, IV, V, and VI) were excluded from the analysis. Altogether, a total of 167 nodules were surgically excised (from the 463 Bethesda class III nodules), giving a malignancy rate, overall, of 27.6% (n = 46), as shown in Figure 2. 

Table 1 lists the factors related to the risk of malignancy (ROM) among the nodules with surgical pathology (n=167). Patients having the following features of TSH levels of >4.5 mIU/L (35%), TN <2 cm (34.6%), solid or nearly solid (28.7%), highly hypoechoic (58.3%), longer more than wide (50%), lobulated (45.5%), punctate echogenic (48.6%), ACR TIRAD 5 (55.2%) and ATA category, high suspicion (50%) displayed a higher ROM value. The chi-square test indicated a remarkable relationship between the malignant and benign nodules, with reference to the echogenicity (*p* = 0.008), echogenic foci (*p* = 0.006), ACR TIRAD (*p* = 0.003) and ATA category (*p* = 0.002). 

**Table 1:** Factors associated with risk of malignancy among surgical pathology (n = 167) ACR, American College of Radiology; ATA, American Thyroid Association; TIRAD, Thyroid Imaging Reporting and Data TSH, thyroid-stimulating hormone

Variables	Number of nodules	Benign (n=121)	Malignant ((n=46)	ROM	p value
Age (years)					
≤45	81	59	22	27.2	0.526
>45	86	62	24	27.9	
Gender					
Female	118	85	33	30	0.505
Male	49	36	13	26.5	
TSH (mIU/l)					
≤0.4	7	5	2	28.6	0.723
0.5-4.5	140	103	37	26.4	
>4.5	20	13	7	35	
Known hypos					
No	126	92	34	27	0.461
Yes	41	29	12	29.3	
Nodule size (cm)					
<2	52	34	18	34.6	0.118
≥2	115	87	28	24.3	
Composition					
Complex	17	14	3	17.6	0.256
Solid or almost solid	150	107	43	28.7	
Echogenicity					
Hypoechoic	52	34	18	34.6	0.008
Isoechoic	103	82	21	20.4	
Very hypoechoic	12	5	7	58.3	
Shape					
Wider than tall	165	120	45	27.3	0.476
Taller than wide	2	1	1	50	
Margin					
Smooth	156	115	41	26.3	0.152
Labulated	11	6	5	45.5	
Echogenic foci					
None	118	91	27	22.9	0.006
Punctate echogenic	37	19	18	48.6	
Microcalcifications	8	8	0	0	
Peripheral interrupted	4	3	1	25	
ACR TIRAD					
TIRAD 2	14	12	2	14.3	0.003
TIRAD 3	65	51	14	21.5	
TIRAD 4	59	45	14	23.7	
TIRAD 5	29	13	16	55.2	
ATA Category					
Very low suspicion	16	13	3	18.7	0.002
Low suspicion	75	61	14	18.6	
Intermediate suspicion	32	25	7	21.8	
High suspicion	44	22	22	50	

Table 2 highlights the outcome of the regression analysis. In comparison with the patients with TI-RADS 2, the ones having categories TIRAD 3 (OR: 1.64), TIRAD 4 (OR: 1.86), and 5 (OR: 13.5) were found to show a higher risk of malignancy. Similarly, in comparison with the ATA category, very low suspicion, those patients with categories low suspicion (OR: 0.231), intermediate suspicion (OR: 0.230), and patients with high suspicion category (OR: 0.28) revealed a greater risk for malignancy. 

**Table 2:** Factors associated with risk of malignancy (Logistic regression) ATA, American Thyroid Association; TIRAD, Thryoid Imaging Reporting and Data

Variables	Variable	OR	(95% CI) Lower-Upper		p value
Echogencity	Hypoechoic	1			
	Isoechoic	0.625	0.088	4.41	0.637
	Very hypoechoic	3.62	0.833	15.7	0.086
Echogenic foci	None	1			
	Punctate echogenic	0.469	0.048	4.55	0.514
	Microcalcifications	0.52	0.031	2.31	0.67
	Peripheral interrupted	0.65	0.809	0.22	0.65
TIRAD	TIRAD 2	1			
	TIRAD 3	1.23	0.708	3.23	0.72
	TIRAD 4	8.75	0.210	0.295	0.259
	TIRAD 5	13.5	0.208	888	0.221
ATA	Very low suspicion	1			
	Low suspicion	0.43	0.041	3.12	<0.001
	Intermediate suspicion	0.84	0.003	2.643	0.159
	High suspicion	0.95	0.026	16.3	0.79

**Figure 3:**
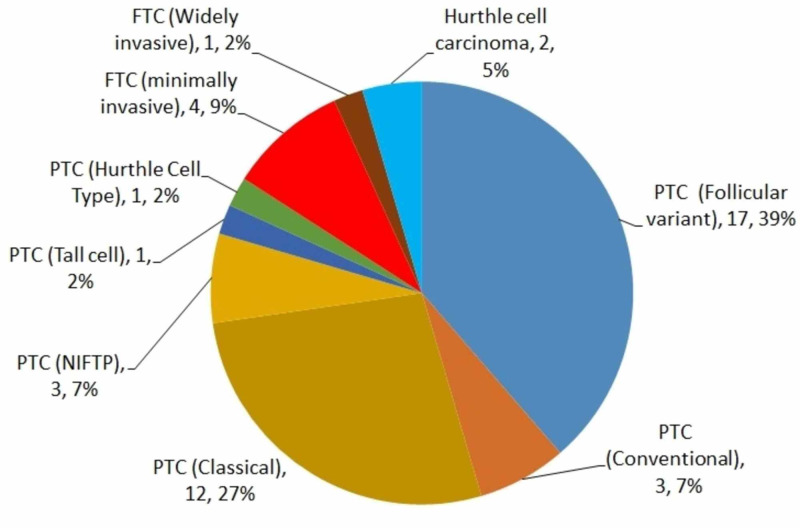
Types of thyroid cancer among the surgical pathology Values are presented as numbers and percentages. PTC, Papillary thyroid carcinoma; FTC, follicular thryoid carcinoma; NIFTP, noninvasive follicular thyroid neoplasm with papillary-like nuclear features

The findings from the study cohort revealed the commonest types of thyroid cancer as the PTC follicular variant (39%) and PTC classical (27%). In the cohort population 3 cases of NIFTP (6.5 %) category were identified. Excluding the NIFTP (n=3), the occurrence of malignancy in the study population was an overall 25.7 % (Figure 3).

Table 3 reveals a comparison of the rates of malignancy (ROM%) after surgical resection for the diagnostic categories of fine-needle aspiration and the risk of malignancy in recent studies. A total of 167 nodules from the 463 Bethesda class III nodules were surgically removed, showing 27.6% (n = 46) malignancy overall; from the 29 repeated Bethesda III, 10 exhibited ROM, with malignancy of 34.4%. 

## Discussion

This study identifies the degree of malignancy risk and related risk factors in the surgical pathology of the Bethesda Category III thyroid nodules at the tertiary care center in Riyadh, Saudi Arabia. The results of the present study revealed that females showed higher ROM, and the male/female ratio for thyroid cancer thus identified (1:4.4) concurs with the understanding that women are more commonly susceptible to thyroid cancer than men; these results are in agreement with earlier reports [11-12]. Further, in an earlier study, it is evident that in women, thyroid cancer ranks seventh in the list of the commonest malignancies; however, it does not rank among the 15 commonest cancers that affect men; it is a fact that differentiated thyroid cancer originating from the follicular cells, such as follicular thyroid cancer and papillary thyroid cancer, occur more commonly in women [11-12]. In another study, among men and women aged over 45 years, TN was found to be prevalent one-third times more often in women than in men [13]. However, earlier studies reported that neither gender nor age did not appear to be a statistically significant carcinoma predictor in these nodules, a fact which corresponds to the findings of the present study [9,14]. 

In this present study, we found that overall, nearly one-third of the surgically excised nodules were found to be malignant (46/167). Further, patients showed higher percentage values for the ROM were those having the following features: TSH values of >4.5 mIU/L (35%), TN <2 cm (34.6%), solid or nearly solid (28.7%), highly hypoechoic (58.3%), longer than wide (50%), lobulated (45.5%), punctate echogenic (48.6%), ACR TIRAD 5 (55.2%) and ATA category, and intermediate suspicion (21.8%). Further, findings from the present study showed a significant relationship with the echogenicity, echogenic foci, ACR TIRAD, and ATA category between the malignant and benign nodules. This showed clear evidence to support that the TN size, TSH, patient age, and gender had no statistical relationship with the ROM. It is important to note that among the malignant patients, the nodule size being <2 cm, nearly 34% revealed malignancy, while 24.3% of the ones in the 1.0 to 1.9 cm diameter range were cancerous. This result was likely because such nodules are nominated for surgical excision, only if they are suspected to be malignant. However, results from the univariate analysis showed that nodule size is reported to have no predictive value of malignancy and therefore is considered an unreliable factor in clinical decision making [15]. Prior research refers to the fact that nodule size may facilitate an assessment of cancer risk, although the data do not concur with the findings of other studies, giving evidence of the absence of correlation between nodule size and risk of malignancy of the TN [9]. Besides, a recent study claimed that nodule size has no correlation with the risk of malignancy, although the link between nodule size and malignancy risk is dependent upon the histological character of thyroid cancer [16]. With reference to the TSH findings, several studies are in agreement that there is no relationship between the TSH level and the risk of malignancy [9,17].

Several studies have indicated the AUS/FLUS diagnostic group has posed an ongoing challenge in terms of clinical management [3,5]. Although several patients with AUS/FLUS BSRTC diagnosis undergo a repeat FNAB, wide research has been done focusing on the application of adjunctive molecular testing to enable suitable clinical management. Strategies like this increase the complexity and cost of patient management. Over the recent years, personalized approaches have been recommended in which all the information available, including clinical, radiologic, and cytopathologic data are used to manage these AUS/FLUS lesions [18]. While earlier reports indicated a 5% to 19% ROM for patients in the AUS/FLUS category, many of them need surgery (usually hemithyroidectomy) before a definitive diagnosis can be made [14,19]. If the final pathology shows malignancy, a second surgery (completion thyroidectomy) is the norm [19]. Therefore, several clinical factors need to be assessed to identify the ROM in AUS/FLUS patients [19]. It is noteworthy that the current study revealed that 10 of the 29 patients with repeated Bethesda III exhibited ROM, which is 34.4% malignancy. Earlier studies gave almost identical values of 37.5% and 33.3%, respectively [20-21]. However, in a recent study, 72 of the 113 patients with repeated Bethesda III revealed ROM, a malignancy rate of 63.7%; however, by way of contrast, one study reported that two out of eight patients with repeated Bethesda III showed ROM, which is 25% malignancy [22]. Reports show that surgically resected AUS/FLUS nodules reveal ROM in the 6% to 48% range, as emphasized in the present ATA guidelines [2]. Likewise, in a meta-analysis performed by comparing 13 different centers after the BSRTC was implemented, the findings showed ROM for AUS/FLUS nodules in the 19% to 38% range; this result concurs with the findings of this study, in which 167 nodules of the 463 Bethesda class III nodules, were surgically removed, indicative of overall malignancy of 27.6% (n = 46) [23]. In addition, three cases of the category noninvasive follicular thyroid neoplasm with papillary-like nuclear features (NIFTP; 6.5%) were identified in the cohort population. These NIFTPs, under ultrasound examination, are usually considered low-suspicion/ pre-malignant nodules [24]. In the FNA cytology, most of the NIFTPs are categorized under indeterminate (AUS/FLUS, FN/SFN, or SM) [24]. Excluding the NIFTP (n=3), the study population showed an overall malignancy of 25.7%.

From the regression analysis done in the current work, a stepwise rise is evident for each category awarded by ACR TI-RADS, and this corresponds to the findings reported in earlier studies. While the ATA categories showed similar findings, the predictions are lower than those made for the ACR TI-RADS category [25]. Besides, the majority of studies reported a higher risk of malignancy than did the TBSRTC for the same category. This variation may perhaps have been due to the heterogeneity of this category, higher risk of malignancy in certain practice settings like the tertiary referral centers, subjective and controversial cytological interpretation, and AUS/FLUS overuse [26]. In the real-world practice, patient-related factors, including a history of prior radiation exposure to neck, or a family history of thyroid cancer, affect the decision to subject the patient to surgery and the extent of that surgery. However, this data is normally unavailable to the reporting cytopathologists. Definitely, further examination of these factors, together with the BSRTC diagnosis, could potentially further enhance the estimation of ROM and cancer and thus properly guide the treatment [27].

The present study identified the PTC follicular variant (39%) and PTC classical (27%) as the commonest forms of thyroid cancer in the study population. Earlier research reported findings that corroborated this result and showed that generally, PTC is the commonest type of thyroid cancer [28]. In fact, 80% of all thyroid malignancies and higher than 90% of the differentiated thyroid cancers are of the PTC variety. A sudden surge in the prevalence of PTC in recent decades has drawn greater attention to the treatment of this disease [29]. The FTC, however, does not have such a high incidence of occurrence, although well-differentiated thyroid carcinomas are on the increase everywhere else, a fact which concurs with the results reported by this study [29].

**Table 3:** Comparison rates of malignancy (%) on surgical resection for fine-needle aspiration diagnostic categories and malignancy risk of recent studies [21-22], [30] AUS, atypia of undetermined significance; FLUS, follicular lesion of undetermined significance; FNAs, fine-needle aspirations; ROM, risk of malignancy

Studies	Published year	AUS/FLS among the whole FNAs	Overall ROM	Repeated Bethesda III (ROM)
Jan et al.	2019	909/29937 (3.1%)	204 (55.6%)	72/113 (63.7%)
Cohen et al.	2017	84/498 (16.8%)	15/44 (34%)	3/8 (37.5%)
Ho et al.	2014	709/8862 (8%)	144 (37.8%)	4/12 (33.3%)
Stanek Widera et al.	2016	395/16656 (2.3%)	8/35 (22%)	2/8 (25%)
Present study	Current	463/4850 (9.6%)	46/167 (27.5%)	10/29 (34.4%)

The present study has some important limitations, which include a substantial loss in follow-up, the study being limited to a single center, limited number risk factors studied, and the lack of using molecular mutation tests as additional diagnostic tools in the prediction of malignancy risk in AUS/FLUS.

## Conclusions

In conclusion, the nodules with AUS/FLUS cytology malignancy rate are comparable with the earlier estimations of other countries. The ACR TI-RAD displayed more accurate diagnostic performances in predicting malignancy in the Bethesda III nodules. However, to confirm the accuracy of the molecular marker tests in specific cytological scenarios, more extensive studies are required in the future.
